# Race/ethnicity and potential suicide misclassification: window on a minority suicide paradox?

**DOI:** 10.1186/1471-244X-10-35

**Published:** 2010-05-19

**Authors:** Ian RH Rockett, Shuhui Wang, Steven Stack, Diego De Leo, James L Frost, Alan M Ducatman, Rheeda L Walker, Nestor D Kapusta

**Affiliations:** 1 Department of Community Medicine and the Injury Control Research Center, PO Box 9190 West Virginia University, Morgantown, West Virginia, 26506, USA; 2 National Institute for Occupational Safety and Health, 1095 Willowdale Road, Morgantown, West Virginia, 26505-2845, USA; 3 Department of Criminal Justice, 2305 FAB, Wayne State University, Detroit, Michigan, 48202, USA; 4 Australian Institute for Suicide Research and Prevention, World Health Organization Collaborating Centre for Research and Training in Suicide Prevention, Griffith University, Mt Gravatt, Queensland, 4122, Australia; 5 Department of Pathology, PO Box 9203, West Virginia University, Morgantown, West Virginia, 26506, USA; 6 Department of Psychology, Psychology Building, University of Georgia, Athens, Georgia, 30602-3013, USA; 7 Medical University of Vienna, Department of Psychoanalysis and Psychotherapy, Waehringer Guertel 18-20, A-1090 Vienna, Austria

## Abstract

**Background:**

Suicide officially kills approximately 30,000 annually in the United States. Analysis of this leading public health problem is complicated by undercounting. Despite persisting socioeconomic and health disparities, non-Hispanic Blacks and Hispanics register suicide rates less than half that of non-Hispanic Whites.

**Methods:**

This cross-sectional study uses multiple cause-of-death data from the US National Center for Health Statistics to assess whether race/ethnicity, psychiatric comorbidity documentation, and other decedent characteristics were associated with differential potential for suicide misclassification. Subjects were 105,946 White, Black, and Hispanic residents aged 15 years and older, dying in the US between 2003 and 2005, whose manner of death was recorded as suicide or injury of undetermined intent. The main outcome measure was the relative odds of potential suicide misclassification, a binary measure of manner of death: injury of undetermined intent (includes misclassified suicides) versus suicide.

**Results:**

Blacks (adjusted odds ratio [AOR], 2.38; 95% confidence interval [CI], 2.22-2.57) and Hispanics (1.17, 1.07-1.28) manifested excess potential suicide misclassification relative to Whites. Decedents aged 35-54 (AOR, 0.88; 95% CI, 0.84-0.93), 55-74 (0.52, 0.49-0.57), and 75+ years (0.51, 0.46-0.57) showed diminished misclassification potential relative to decedents aged 15-34, while decedents with 0-8 years (1.82, 1.75-1.90) and 9-12 years of education (1.43, 1.40-1.46) showed excess potential relative to the most educated (13+ years). Excess potential suicide misclassification was also apparent for decedents without (AOR, 3.12; 95% CI, 2.78-3.51) versus those with psychiatric comorbidity documented on their death certificates, and for decedents whose mode of injury was "less active" (46.33; 43.32-49.55) versus "more active."

**Conclusions:**

Data disparities might explain much of the Black-White suicide rate gap, if not the Hispanic-White gap. Ameliorative action would extend from training in death certification to routine use of psychological autopsies in equivocal-manner-of-death cases.

## Background

Suicide kills approximately one million people annually [1] and over 30,000 Americans [2]. Analysis of this leading public health problem in the United States is complicated by undercounting at local [3] and state levels [4]. While (non-Hispanic) Blacks and Hispanics, the major racial/ethnic minorities, face many socioeconomic and health disparities, their suicide rates are reported as less than half the rate for (non-Hispanic) Whites [2].

Case ascertainment of suicide is inferior to that of homicide, with training of law-enforcement officials who conduct death-scene investigations "geared towards the confirmation or denial of foul play" [5]. Classifying a probable suicide as suicide requires strong corroborative evidence, such as a suicide note, psychiatric history, biological and toxicological evidence, and testimony from reliable witnesses [6,7]. Impediments include religious proscriptions and cultural taboos against suicide, concerns about precipitation of familial stigma, awareness that life insurance companies may resist redemption of policies on suicide decedents, political pressure, threatened or actual litigation, and a paucity of psychological autopsies [8-10]. Most official suicides are probably true suicides [11,12], and suicide appears more susceptible than homicide or unintentional injury death to misclassification under injury of undetermined intent [11,13].

Poor mental health is the common proximal determinant of suicide [14,15], but data gaps can hamper official investigations of psychiatric problems in equivocal manner-of-death cases [16]. Approximately one-half of suicides have been diagnosed with a mental disorder and received therapy from a mental health professional [12]. Yet, psychiatric comorbidity is documented in less than 10 percent of their death certificates [17], with lower prevalence in Blacks and Hispanics compared to Whites [18]. If reflecting a similar relative gap in medical examiner and coroner knowledge and records, a racial/ethnic discrepancy in the prevalence of this comorbidity reported on death certificates could represent a contributing factor in the suicide rate gap.

For the period 2003-2005, annualized national crude and age-adjusted suicide rates (based on the US 2000 standard population) for Whites were 13.5 and 12.8 per 100,000 population, respectively, compared to 5.3 and 5.4 for Blacks and 5.2 and 5.7 for Hispanics [2]. Comparative mental health status and treatment profiles alone make this rate gap paradoxical. We conservatively assume that the proximate physical and mental health status of the minority suicides was at least as poor as that of White suicides, an assumption supported by the Sample Adult component of the 1998-2003 National Health Interview Surveys, which revealed worse physical health and more unmet mental healthcare needs for Blacks and Hispanics [19]. Recent national surveys indicate equal or higher prevalence of depression among those racial/ethnic minorities compared to Whites [20,21]. Blacks and a sub-group of Hispanics, Mexican-Americans, were also less likely to receive depression care, particularly guideline-concordant care [22].

Although confined to Blacks and Whites (Hispanics not independently presented), data from a Tennessee records-based study addressed discrepant documentation of psychiatric comorbidity between US minority and White suicides [23]. Subjects were adult enrollees in TennCare, Tennessee's Medicaid-waiver managed-care program, which is intended to offer equal financial access to all low-income groups. Only 29% of Black suicides and possible suicides, as compared to 51% of White counterparts, used prescribed antidepressants in the year prior to death. Study investigators interpreted this differential as indirect evidence that serious mood disorders are underdiagnosed or undertreated in Blacks. However, they acknowledged that this group may have received more non-pharmacological therapy than whites, and thus a relatively smaller proportion may have had antidepressant prescriptions filled. Salient to our subsequent operationalization of potential suicide misclassification, they operationalized possible suicides as subjects whose underlying cause of death was officially classified as injury of undetermined intent. These investigators also performed a sensitivity analysis excluding these decedents. Findings approximated their primary ones.

Assuming that diagnostic recognition and consequent intervention can prevent suicide, social groups with access, diagnostic, or treatment deficits should be susceptible to excess suicide risk and rates. Comparative racial/ethnic suicide data appear to discredit this expectation. However, to the degree that mentally distressed members of a social group are less apt than others to have accessible psychiatric records, death investigators may be less likely to find evidence of psychopathology or suicidal intent and more likely to rule manner of death as undetermined.

Empirical research supports the possibility that Blacks are more misclassification-prone than Whites. For example, a 1970s New York City study of medical examiner data estimated that suicide was underenumerated by 80% for Blacks compared to 42% for Whites [3], and is consistent with other evidence that variation in suicide misreporting between Blacks and Whites is systematic [4,24]. While a paradoxical advantage in general health has been observed for Hispanics [25], a state-based study indicates that race may outweigh ethnicity in understanding variable suicide data quality [26].

Gender, age, and social class could be determinants of suicide misclassification. Females demonstrate a greater proclivity than males to employ less violent, "less active," and more equivocal methods of suicide [27]. Regarding age, medicolegal authorities may be more reluctant to attribute suicidal intent to deaths of adolescents and younger adults than those of older adults [28]. Although a Scottish study found no association between social class and undetermined death classification [29], we have not detected any research which examined this relationship in the United States.

Trying to comprehend a racial/ethnic gap in suicide rates, we sought to assess whether race/ethnicity and other decedent characteristics were associated with potential suicide misclassification.

## Methods

This cross-sectional study accessed underlying cause-of-death data for the period 2003-2005 from WISQARS, the Web-based Injury Statistics Query and Reporting System, and corresponding de-identified multiple-cause-of-death (MCOD) data from the National Center for Health Statistics (NCHS) public-use files. Causes of death were precoded under the *International Statistical Classification of Diseases and Related Health Problems, Tenth Revision ICD-10 *[30] for the 105,946 White, Black, and Hispanic residents of the 50 US states and the District of Columbia whose manner of death was either suicide, operationalized as death from intentional self-harm (ICD-10 X60.0-X84.9) or its sequelae (Y87.0), or death from injury of undetermined intent (ICD-10 Y10-Y34) or its sequelae (Y87.2, Y89.9). We excluded other racial/ethnic groups due to heterogeneity and small numbers. Restricted to persons dying at ages 15 years and older, our study population accounted for 99% of all official suicides during the observation period [2].

Distinguishing race/ethnicity and manner-of-death, we first profiled our study population according to gender, age (15-34; 35-54; 55-74; 75+ years), education (0-8 years; 9-12 years; 13+ years; unknown) as a proxy for social class, documentation of psychiatric comorbidity on the death certificate (no/yes), and injury mode or method (less/more active). Unknowns were incorporated into our education variable as a separate category because they represent deficient data and are numerous.

We operationalized psychiatric comorbidity on the death certificates as follows: schizophrenia (ICD-10 F20-29), depression/mood disorders (F30-39), and other non-organic mental health disorders (F40-99). Both an Australian MCOD study [31] and its US replication [17] showed positive associations between these disorders and suicide. We categorized "more active" (violent) modes of death as ICD-10 injury manner and mechanism codes: X70 intentional self-harm by hanging, strangulation and suffocation; X72 intentional self-harm by handgun discharge; X73 intentional self-harm by rifle, shotgun and larger firearm discharge; X74 intentional self-harm by other and unspecified firearm discharge; X78 intentional self-harm by sharp object; X80 intentional self-harm by jumping from a high place; Y20 hanging, strangulation and suffocation, undetermined intent; Y22 handgun discharge, undetermined intent; Y23 rifle, shotgun and larger firearm discharge, undetermined intent; Y24 other and unspecified firearm discharge, undetermined intent; Y28 contact with sharp object, undetermined intent; and Y30 falling, jumping or pushed from a high place, undetermined intent. This formulation reflected Scottish observations that mortality from hanging, shooting, cutting, and jumping from a height generated the highest likelihoods that decedents from a pool of suicides and undetermined deaths would be classified as suicides [29]. We categorized residual injury deaths as "less active."

We performed unconditional multiple logistic regression analyses on pooled undetermined and suicide deaths to estimate associations using SAS (version 9.13, Cary, NC). Our outcome measure, potential suicide misclassification, was a binary variable where 1 = undetermined injury intent and 0 = suicide. We assumed that suicides comprised true suicides^11 ^and undetermined deaths a high proportion of misclassified suicides [13,32]. Statistical analyses involved four models. The first model contained a single predictor, race/ethnicity, and the second comprised race/ethnicity, age, gender, and education. The third model added psychiatric documentation, and our full and final model also incorporated injury mode or method.

## Results

**Figure 1:**
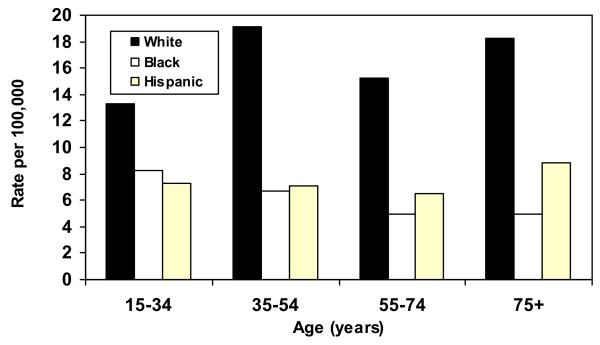
Suicide rates by race/ethnicity and age: United States, 2003-2005.

**Figure 2:**
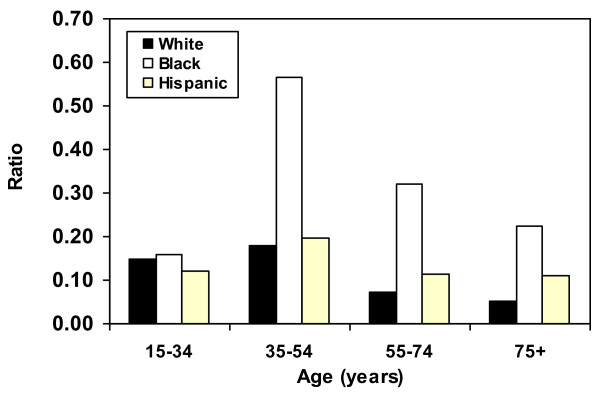
Rate ratio of mortality of undetermined injury intent to suicide by race/ethnicity and age: United States, 2003-2005

Suicide rates were universally much higher for Whites than for Blacks and Hispanics at all ages, and the gap was more pronounced after age 35 (Figure 1). Suicide rates for Blacks and Hispanics were similar except among older decedents. Comparisons of age-specific ratios of undetermined injury deaths to suicide deaths revealed radically different patterns (Figure 2). Blacks exhibited a much higher ratio after age 34, an inversion of the suicide rate pattern. Generally, Hispanic ratios resembled White ratios much more than those of Blacks.

Table 1 profiles the study population differentiating race/ethnicity and manner-of-death. Male predominance was comparatively diminished for undetermined deaths and when decedents were white. Suicides were older than the undetermined. Any psychiatric documentation was uncommon in the undetermined category and highest among White suicides. Hispanics registered the lowest prevalence of a less active mode of injury independent of manner of death.

**Table 1:** Decedent characteristics by race/ethnicity and manner of death, United States, 2003-2005

		White		Black		Hispanic	
		
Characteristic		Suicide (n = 80,235)	Undetermined* (n = 10,962)	Suicide (n = 5,707)	Undetermined* (n = 1,856)	Suicide (n = 6,276)	Undetermined* (n = 910)
**Male**		79.2%	61.0%	82.0%	69.2%	84.5%	72.1%
**Age **(years)							
	15-34	25.1%	27.2%	49.0%	23.8%	50.9%	41.9%
	35-54	43.2	57.2	35.9	62.3	34.4	46.6
	55-74	21.4	11.6	11.7	11.5	11.0	8.7
	75+	10.3	4.0	3.4	2.4	3.8	2.9
**Education **(years)							
	0-8	4.0%	4.0%	3.8%	4.7%	18.9%	16.8%
	9-12	55.0	60.7	59.9	68.7	58.0	58.1
	13+	36.1	28.8	27.2	18.2	19.1	15.9
	unknown	4.9	6.5	9.1	8.5	4.0	9.0
**Psychiatric Documentation**							
	none	93.0%	97.2%	96.8%	98.8%	97.3%	97.8%
**Mode of Injury**							
	less active**	22.3%	92.9%	19.0%	92.5%	16.6%	85.2%

* Injury of undetermined intent.

** Deaths classified under ICD-10 X60-69, X71, X75-77, X79, X81-84, Y10-19, Y21, Y25-27, Y29, and Y31-34. Residual injury deaths were classified as more active.

Table 2 reports logistic regression results. In Model 1, where race/ethnicity was the sole criterion variable, Blacks appeared more than twice as prone to potential suicide misclassification as Whites. Model 2 incorporated remaining demographics which slightly reduced the Black excess. Females were over twice as prone to potential misclassification as males, and education was negatively associated with such proneness. In Model 3 Hispanics, as well as Blacks, manifested excess potential for suicide misclassification, and only decedents aged 55-74 showed less potential than the youngest. Decedents without psychiatric documentation were almost seven times more likely than opposites to be classified as undetermined. In Model 4, decedents in the "less active" group were 46 times as likely to be classified as undetermined as "more active" counterparts. Accounting for mode of injury death increased potential for suicide misclassification for Blacks while decreasing it for Hispanics. The youngest showed greater potential for misclassification than their elders, and the gender effect was nullified in the face of the injury mode-gender nexus.

**Table 2:** Logistic regression models of the association between race/ethnicity, other individual characteristics, and potential suicide misclassification,* United States, 2003-2005

Characteristic	Model 1	Model 2	Model 3	Model 4
	**(n = 105,946)**	**(n = 105,946)**	**(n = 105,946)**	**(n = 105,946)**
**Race/Ethnicity**	**OR (95% CI)**	**OR (95% CI)**	**OR (95% CI)**	**OR (95% CI)**
White (referent)	1.00	1.00	1.00	1.00
Black	2.38 (2.25, 2.52)	2.26 (2.13,2.39)	2.28 (2.21, 2.36)	2.38 (2.22, 2.57)
Hispanic	1.08 (1.00, 1.16)	1.00 (0.93,1.08)	1.28 (1.24, 1.32)	1.17 (1.07, 1.28)
**Gender**				
male (referent)		1.00	1.00	1.00
female		2.36 (2.27,2.45)	2.29 (2.25, 2.34)	1.01 (0.97, 1.06)
**Age **(years)				
15-34 (referent)		1.00	1.00	1.00
35-54		1.40 (1.34,1.46)	1.37 (1.34, 1.40)	0.88 (0.84, 0.93)
55-74		0.61 (0.58,0.66)	0.85 (0.82, 0.87)	0.52 (0.49, 0.57)
75+		0.44 (0.40,0.48)	1.47 (1.42, 1.52)	0.51 (0.46, 0.57)
**Education (years)**				
0-8		1.82 (1.66,2.00)	1.82 (1.75, 1.90)	2.34 (2.10, 2.62)
9-12		1.51 (1.45,1.58)	1.43 (1.40, 1.46)	1.66 (1.58, 1.74)
13+ (referent)		1.00	1.00	1.00
unknown		1.84 (1.70,1.99)	1.37 (1.32, 1.43)	2.08 (1.89, 2.28)
**Psychiatric Documentation**				
yes (referent)			1.00	1.00
no			6.56 (6.16, 6.99)	3.12 (2.78, 3.51)
**Mode of Injury**				
more active (referent)				1.00
less active**				46.33 (43.32, 49.55)

* Potential suicide misclassification was operationalized as a binary outcome measure of manner of death: undetermined intent (1) and suicide (0).

** Deaths classified under ICD-10 X60-69, X71, X75-77, X79, X81-84, Y10-19, Y21, Y25-27, Y29, and Y31-34. Residual injury deaths were classified as more active.

## Discussion

This examination of national MCOD data identifies probable health-data disparities across a range of decedent characteristics. Controlling for demographics and other key determinants, suicide data quality for Blacks, and to a smaller extent for Hispanics, seems inferior to that for Whites. We estimated that Blacks were over twice as prone to potential suicide misclassification as Whites and Hispanics 17% more prone. Excess potential for misclassification was also indicated for youth and the lesser educated. Results may support a previously proposed but understudied hypothesis that medicolegal authorities are more hesitant to attribute suicidal intent in death investigations involving the young [28]. We further estimated that decedents with no mention of psychiatric comorbidity on their death certificates carry a three-fold greater potential for suicide misclassification than opposites. Although the association between mode of injury and potential misclassification was marked, incorporating injury mode into the multivariate analysis nullified only the gender association. Our findings confirm that documentation of psychiatric comorbidity on US death certificates is deficient [17] and that deficits are magnified among the two major racial/ethnic minorities [18]. General deficits in death certificates and clinical training in death certification have been widely reported [33-35], and training problems extend to medical examiners and coroners [10]. Despite low prevalence, factoring in psychiatric documentation into our study suggested excess potential for suicide misclassification for Hispanics as well as for Blacks.

Confined to death certificate data, our study only indirectly assessed the relationship between race/ethnicity, other decedent characteristics, and suicide misclassification, a formidable target. A second and related limitation is that representation of true suicides among undetermined injury deaths, our proxy for misclassified suicides, likely varies by decedent demographics including race/ethnicity [36]. A strong mitigating factor was our control for mode, method, or mechanism of injury death. While undetermined intent deaths comprise the category most susceptible to misclassification, other mortality categories can also obfuscate suicides [4]. Some dwarf that category, notably ill-defined and unknown causes and unintentional poisonings [2,37,38]. These and other categories, such as "suicide-by-cop" [24], subsumed under subject-precipitated homicide, all possibly contribute to differential suicide data quality between Blacks, Hispanics, and Whites. Our analysis focused on the undetermined intent category because the evidence base for its inclusion remains stronger in comparison to other categories [11,13]. Nonetheless, the issue of differential susceptibility to suicide misclassification by cause of death warrants in-depth research.

A third study limitation, but selectively problematic owing to small numbers, our results might have been modified had we been able to factor in uncontrolled heterogeneity inherent in all three racial/ethnic groups. A potentially important confounder is the immigration status of decedents. However, beyond the constraint of small numbers, the MCOD files lack data on duration of residence in the United States and on country of origin. Implicating the acculturation process, future research might address the impact of these variables since they could relate to within and across group assessment of risk for both suicide and suicide misclassification.

Since suicide case ascertainment is typically local, a fourth study limitation is our omission of contextual factors. A focus for our future research, these factors include community attitudes and actions in response to suicide, as well as poverty, discrimination, segregation, healthcare, and type and trust of the medicolegal system.

Ideally we would have had access to medical examiner and coroner records on psychiatric histories of our study population. We found that a proxy for these data, absence of documentation (versus documentation) of psychiatric comorbidity on the death certificate, was a strong predictor of potential suicide misclassification. And the very low prevalence of this documentation, taken at face value, might suggest that official knowledge or lack of knowledge of the psychiatric history of decedents is a minor contributor to differential misclassification by race/ethnicity. We appreciate that these officials likely know much more about such history than is reflected on the death certificate. Nevertheless, germane to preliminary comprehension of the black-white suicide paradox, we had no a priori reason to believe that the nature and magnitude of the relative racial comorbidity gap manifest from death certificates would not mirror a similar gap in medical examiner and coroner records. However, this remains an empirical question.

The potential of a social group to have their suicides misclassified is related to the extent to which members leave a suicide note, a key piece of forensic evidence for classifying a death as suicide [39,40]. In a preliminary analysis of 2003-2006 data from the National Violent Death Reporting System, a system currently confined to less than 20 states [41], we determined that 31% of white suicides left a note, but only 18% of black suicides and 21% of Hispanic suicides. We suspect that this differential in forensic evidence contributes to misclassification-proneness and the suicide paradox.

Risk factors collectively predict much higher suicide rates for Blacks than their official rates. On the other hand, although not yet subjected to rigorous testing, countervailing forces may diminish their suicide risk relative to Whites. Blacks manifest higher religiosity and less cultural acceptance of suicide [42,43]. Some researchers suggest that Blacks possess a preference for externalizing extreme aggression as homicide rather than internalizing it as suicide [44], an ad hoc explanation we find uncompelling. Indeed, no research explains how being Black protects against suicide, especially when viewed in context with other violent behaviors like homicide [45]. Moreover, cultural factors within Black communities could foster relative under-documentation of psychiatric comorbidity on the death certificates of their suicides. Arguing for the value of sociocultural autopsies in comparative racial/ethnic suicide research [46], these factors include greater self-stigma and public stigma which these communities attach to mental illness and treatment compared to White communities [47].

## Conclusions

Due to the apparent links between decedent characteristics and potential suicide misclassification, we hypothesize that health-data disparities explain much of the Black-White paradox. Support is weaker for a kindred hypothesis about the Hispanic-White paradox. Harboring adverse implications for treatment, policy, and prevention, health-data disparities are less transparent than health disparities [48]. Our findings identify a need for interdisciplinary study of suicide data disparities in both clinical and nonclinical settings. They motivate our call for expanding equivocal-manner-of-death investigations; standardizing protocols for death investigations; conducting psychological autopsies in equivocal cases; better integrating mortality data collection and reporting; establishing prevention programs where a latent clinical problem exists that is amenable to therapy; and routinizing training in death certification for medical students, medical residents, practicing physicians, medical examiners, and coroners.

Future research must revisit the strong association apparent between low education and potential for suicide misclassification. Since persons with fewer than nine years of education generally experience low socioeconomic status, a social class analysis may yield insights about processes which even transcend racial/ethnic disparities. Among important variables to incorporate in a class analysis of suicide misclassification would be religion, religiosity, race, ethnicity, nativity, income, education, occupation, and employment status, as well as healthcare nature, quantity, quality, access, and utilization.

## Competing interests

The authors declare that they have no competing interests.

## Authors' contributions

IRHR conceived and designed the study. IRHR and SW obtained, prepared, and managed the data, and performed the statistical analyses. IRHR conducted the literature review. IRHR, SS, DD, JLF, AMD, RLW, and NDK interpreted the findings, and drafted the manuscript. All authors read and approved the final manuscript.

## Pre-publication history

The pre-publication history for this paper can be accessed here:

http://www.biomedcentral.com/1471-244X/10/35/prepub
